# Acute Appendicitis in Children During War Conflict: Results from a Multicenter Study

**DOI:** 10.3390/jcm14134615

**Published:** 2025-06-29

**Authors:** Gal Becker, Igor Sukhotnik, Nadav Slijper, Dana Zezmer, Vadim Kapuller, Alon Yulevich, Yair Ben Shmuel, Audelia Eshel Fuhrer, Haguy Kammar, Lili Hayeari, Osnat Zmora

**Affiliations:** 1 Department Pediatric Surgery, Dana-Dwek Children’s Hospital, Tel Aviv Sourasky Medical Center, Tel Aviv 6423906, Israel; becker.gal@gmail.com (G.B.); igorsu@tlvmc.gov.il (I.S.); haguyk@tlvmc.gov.il (A.E.F.); kammarh@gmail.com (H.K.); 2 Faculty of Medical and Health Sciences, Tel Aviv University, Tel Aviv 6997801, Israel; 3 Department Pediatric Surgery, Galilee Medical Center, Nahariya 2210001, Israel; nadav.slijper@gmail.com; 4 Department Pediatric Surgery, Assuta Ashdod Medical Center, Ashdod 7747629, Israel; dana.zezmer@gmail.com (D.Z.); vadimk@assuta.co.il (V.K.); 5 Department Pediatric Surgery, Ziv Medical Center, Safed 1311001, Israel; alony@ziv.gov.il; 6 Department Pediatric Surgery, Bnai Zion Medical Center, Haifa 3339419, Israel; yairbenshmuel@gmail.com (Y.B.S.); lili.hayari@gmail.com (L.H.); 7 Department Pediatric Surgery, Shamir Medical Center, Zerifin 7073001, Israel

**Keywords:** appendicitis, war, children

## Abstract

**Background/Objectives:** War conflicts impact public health and patient hospital presentations. We aimed to assess the incidence and severity of acute appendicitis (AA) in children during the 2023 Israeli–Hamas–Hezbollah war. **Methods:** This multicenter retrospective cohort study included children (<18 years) admitted with AA in six medical centers in a 2-month period during the war (7 October–30 November 2023) and a comparable period in 2022. Demographic, clinical, laboratory, imaging, treatment, and outcome data were collected at individual medical centers and analyzed, with subgroup analysis based on proximity to conflict zones. Statistical tests used were Kolmogorov–Smirnov test, Student’s *t*-test, Mann–Whitney U, and Pearson chi square. *p* < 0.05 was considered significant. **Results:** Among 209 patients (106 in 2023, 103 in 2022), a higher rate of complicated AA during wartime was observed, although not statistically significant (27% vs. 18%, *p* = 0.11). The median symptom-to-presentation time remained 24 h (*p* = 0.64). The overall incidence of AA decreased by 20% in medical centers near conflict zones but increased by 28% in centers distant from conflict zones. The proportion of complicated AA doubled during the war in hospitals close to conflict zones as compared to during pre-war time (16% vs. 9%, respectively, *p* = 0.016), with a trend toward higher C-reactive protein (CRP) levels [26.5 (5.3–107.0) vs. 13 (3.4–40.9), respectively, *p* = 0.075], although symptom-to-presentation times remained unchanged (24 h in both groups, *p* = 0.32). **Conclusions:** Proximity to war zones was associated with an increase in the rate of complicated appendicitis in children. While the causes remain unclear, this finding highlights the complex impact of war on healthcare in general and on the well-being of children in particular.

## 1. Introduction

War is considered a public health emergency as it carries both immediate and long-term physical health consequences. Civilians can be killed or injured directly from explosives or terrorist attacks [1], develop secondary health problems such as emotional and behavioral difficulties, or suffer from scarce access to adequate healthcare [2,3]. War-associated population displacement, compromised transportation, poor environmental conditions, deprivation of necessities (e.g., food, shelter, and water), and the disruption of both curative and preventive health services may impact the management of many diseases. Although war can affect people at any age, children are probably most profoundly affected. The effects of war on children are devastating and long-lasting [2]. In 2011, child deaths constituted approximately 9% of war-related deaths in Syria, and five years later, this increased to over 23% [4].

Acute appendicitis (AA) is the most common surgical emergency affecting children and young people. Several factors may cause changes in the management and outcome of children with AA during war conflicts. These include delayed transfer of children to an appropriate facility for an appendectomy due to the military environment, diversion of pediatric healthcare staff and adult caregivers due to military recruitment, changes due to operating room availability that also meet the essential needs of war victims, and emotional and behavioral problems, which may mask symptoms and cause delays in diagnosis [5]. Presentation delays could also be attributed to fear of leaving home, compromised transportation, and population displacement, resulting in restricted access to primary health services [6] that may result in higher rates of complicated appendicitis.

The Israel–Hamas War (military conflict between Israel and Palestinian Hamas terrorists) began with a Hamas attack on southern Israel on 7 October 2023. On 8 October 2023, Hezbollah, a Lebanese Shia Islamist political party and terrorist group, joined Hamas and attacked northern Israel. In Israel, over 126,000 civilians from both northern Israel and southern Israel were evicted from their homes and transferred to central regions.

The aim of this study was to determine patterns of AA in the pediatric population throughout the country of Israel during the acute and intense first 2 months of this war conflict, as compared to pre-war conditions.

## 2. Materials and Methods

### 2.1. Study Design

We conducted a multicenter retrospective cohort study that included six medical centers, three located close to conflict zones that were directly impacted by the hostilities and three distant from conflict zones. Children who were admitted with AA during the first 2 months of the Israel–Hamas–Hezbollah war conflict (war group) were compared to children who were admitted with AA during the same time period a year earlier (pre-war group). We calculated the required sample size to obtain 90% statistical power. Based on previous publications [7,8], we assumed a 25% rate of complicated appendicitis in the control group (pre-war) and hypothesized that this rate would double to 50% during the war. The calculated sample size was 154 patients in total, with 77 patients per group. Based on the monthly AA admission rate, we assessed that a period of 2 months would provide a sufficient sample size for this study.

### 2.2. Participants

Inclusion criteria: We included all children under age 18 years who were admitted with AA during the 2 months between 7 October and 30 November 2023 and all children who were admitted with AA during the same time period a year earlier, during 7 October –30 November 2022. Only patients in whom imaging supported the diagnosis of AA were included. Sonographic diagnostic criteria were an appendiceal diameter of ≥7 mm, wall hyperemia, and incompressibility.

Exclusion criteria: Patients in whom the diagnosis of AA was uncertain or based on clinical examination alone were excluded from the study, even if treated similarly. Patients whose treatment differed from the standard protocol were excluded as well. A total of two patients were excluded.

Six medical centers from different regions of Israel participated in this study, divided into two groups according to proximity to conflict zones (straight line distance). Medical centers “close to conflict zones” were defined as those located less than 30 km from a conflict zone, and medical centers “distant from conflict zones” were defined as those located more than 30 km away from a conflict zone. Centers “close to conflict zones” included Galilee Medical Center, Nahariya, approximately 10 km from the Lebanese border (*n =* 46); Ziv Medical Center, Safed, approximately 15 km from the Lebanese border (*n* = 24); and Assuta Ashdod Medical Center, approximately 25 km from the border with the Gaza strip (*n =* 25). Centers “distant from conflict zones” included Tel Aviv Sourasky Medical Center, Tel Aviv, approximately 100 km from the Lebanese border and 65 km from the border with the Gaza strip (*n =* 44); Bnai Zion Medical Center, Haifa, approximately 40 km from the Lebanese border and 120 km from the border with the Gaza strip (*n =* 18); and Shamir Medical Center, Be’er Ya’akov, approximately 120 km from the Lebanese border and 40 km from the border with the Gaza strip (*n =* 52).

The participating medical centers received a data sheet with study inclusion and exclusion criteria and the requested information, ensuring that the same units and definitions were used across all participating medical centers. The data were collected and anonymized at each medical center separately by a local physician and transferred to the principal investigator. Data from all participating centers were compiled together in a single data sheet and subjected to a quality review to ensure consistency. Any inconsistencies were clarified with the individual medical center. Statistical analysis was performed on the final unified data.

### 2.3. Variables

We collected demographic, clinical, diagnostic, and treatment data. Categorical variables included gender, presence of specific symptoms, presence of specific physical examination findings, treatment protocol [surgery versus nonoperative management (NOM)], the presence of postoperative complications, and the presence of AA recurrence. Continuous variables included age, laboratory results, American Association for the Surgery of Trauma (AAST) appendicitis imaging grade, AAST appendicitis operative grade, Pediatric Appendicitis Score (PAS) [9], length of stay (LOS), days of postoperative antibiotic treatment, and rate of readmission. Duration of follow-up was defined as the time elapsed between the time of treatment and the time of data collection. Table 1 presents the AAST appendicitis score grading system [8,10].

### 2.4. Definitions

Nonoperative management consisted of antibiotic treatment, with no interventional procedures. The antibiotic regimen consisted of either IV amoxicillin/clavulanic acid or ceftriaxone and metronidazole during hospital stay, followed by 1 week of PO amoxicillin/clavulanic acid.

Complicated appendicitis was determined by either operative or imaging AAST grade ≥ 2.

### 2.5. Primary and Secondary Outcomes

The primary outcome of the study was severity of appendicitis, measured as the percentage of patients who presented with complicated appendicitis and as the mean AAST score per group.

Secondary outcomes included time from presentation to arrival at the emergency department (ED), LOS, and NOM failure.

### 2.6. Ethical Considerations

Approval from local ethics committees was received from all centers. Approval numbers and dates are as follows: Tel Aviv Sourasky Medical Center, TLV-0753-23, 28 December 2023; Galilee Medical Center, NHR-0042024, 16 April 2024; Assuta Ashdod Medical Center, 0044-25-AAA, 2 April 2025; Ziv Medical Center, 0004-24-ZIV, 9 January 2024; Bnai Zion Medical Center, 0028-25-BNZ, 10 April 2025; and Shamir Medical Center 0051-24-ASF, 14 March 2024.

Informed consent was waived as this was a retrospective study based on medical records only, without changes in treatment, and it did not contain any identifying patient data.

### 2.7. Statistical Analysis

Normal distribution of continuous variables was tested by Kolmogorov–Smirnov test. According to the results of this test, we used mean and standard deviation as descriptive statistics and Student’s *t*-test to compare the groups for normally distributed parameters. For non-normally distributed continuous variables, we used median and interquartile range as descriptive statistics and a Mann–Whitney U test to compare the groups. Categorical variables were described as percentages and compared with Pearson chi square. We compared the war group vs. the pre-war group and performed subgroup analysis to compare pre-war vs. war groups within medical centers close to conflict zones and medical centers distant from war zones separately. *p* < 0.05 was considered significant. Statistical Package for the Social Sciences (SPSS), version 29.0 (IBM Corp., Armonk, NY, USA) was used for all statistical analyses.

This study adheres to the STROBE guidelines for the proper reporting of observational studies [11].

## 3. Results

### 3.1. Study Population

Two hundred and nine patients from six medical centers were included in this study. Patients were divided according to their admission period: 106 in group A (war group) and 103 in group B (pre-war group). Patients were not transferred between medical centers during the war. The two groups were similar in terms of patient age (10.7 ± 3.8 years old in group A, 11.4 ± 3.6 in group B; *p* = 0.17) and gender (56% male in group A vs. 67% in group B; *p* = 0.093).

### 3.2. War vs. Pre-War in the Entire Study Population

Table 2 summarizes characteristics of patients in group A (war) versus patients in group B (pre-war) across all participating centers. Time from onset of symptoms to arrival at the ED was similar between the two groups, with a median of 24 h in both groups (*p* = 0.64). The percentage of patients who arrived 48 h or later after the onset of symptoms was also similar between the two groups (23.6% in group A and 23.3% in group B; *p* = 0.96). The proportion of complicated appendicitis was higher in the war group as compared to the pre-war group. However, the difference was not statistically significant (26.7% vs. 17.5%, *p* = 0.11). Among patients diagnosed with complicated appendicitis, right lower quadrant pain was reported in 17 out of 25 patients (68%) in group A and 8 out of 16 patients (50%) in group B (*p* = 0.33). Migration of pain was reported in 7 out of 23 patients (30%) in group A and 4 out of 13 patients (31%) in group B (*p* = 1.00). Anorexia was present in 15 out of 23 patients (65%) in group A as compared to 9 out of 13 patients (69%) in group B (*p* = 1.00). Nausea or vomiting was documented in 20 out of 25 patients (80%) in group A and 15 out of 16 patients (94%) in group B (*p* = 0.38). Fever > 38 °C was documented in 10 out of 25 patients (40%) in group A and 8 out of 16 patients (50%) in group B (*p* = 0.75). None of these differences reached statistical significance. Laboratory results were similar between the groups, including white blood cells (WBCS) and neutrophil counts. C-reactive protein (CRP) was higher in the war group as compared to the pre-war group; however, the difference was not statistically significant.

Table 3 summarizes treatment and outcome parameters in war vs. pre-war groups across all participating centers. There was no difference in the likelihood of NOM for non-complicated AA between the two groups. Of those treated nonoperatively, there was a higher NOM failure rate in the war group; however, the difference was not statistically significant. Average LOS, rate of readmission, and rate of postoperative complications were similar in both groups.

### 3.3. Subgroup Analysis According to Proximity to Conflict Zones

A subgroup analysis according to proximity to conflict zones was performed. Overall, 95 patients were treated in medical centers close to conflict zones (Table 4), and 114 were treated in medical centers distant from conflict zones (Table 5). A 20% decrease in the incidence of AA was observed in medical centers located close to conflict zones from where the government evicted the population, from 53 cases pre-war to 42 cases during the war. Concurrently, a 28% increase was observed in medical centers distant from conflict zones, from 50 cases pre-war to 64 cases during the war.

The proportion of complicated AA in medical centers close to conflict zones doubled during wartime as compared to pre-war time (from 17% pre-war to 39.0% during the war, *p* = 0.016). The proportion of complicated appendicitis in medical centers distant from conflict zones was similar at wartime as compared to pre-war time (18.0% pre-war and 18.8% during war, *p* = 0.92). No other variables were found to be statistically different between pre-war time and wartime in the separate subanalysis groups. However, although not statistically significant, a higher CRP level during war as compared to pre-war was documented in patients who were treated in medical centers close to conflict zones (26.5 mg/L during war as compared to 13 mg/L pre-war, *p* = 0.075).

## 4. Discussion

In our study, we found that in the entire population, the incidence of AA and duration of symptoms before arrival at the ED were similar during wartime as compared to pre-war time, with a trend toward increased proportion of complicated appendicitis during war. More importantly, in a subgroup analysis based on the proximity of the medical center to conflict zones, we found that the rate of complicated appendicitis doubled during the war only in medical centers close to conflict zones, along with a trend toward increased CRP levels in this population. The subgroup analysis is a unique feature of our study, which may provide an internal control group, i.e., the population in centers more distant from war zones, albeit evaluated in the same country/health system and at the same time.

The subgroup analysis also revealed a decrease in the overall incidence of AA in medical centers close to conflict zones, with a reciprocal increase in medical centers distant from conflict zones. This finding may be attributed to the government-ordered evacuation of populations from conflict zones. It might also be that parents sought to place their children in a hospital located as far from the conflict as possible.

In recent years, several studies have reported on the effects of the COVID-19 pandemic on the presentation, management, and outcomes of AA in general, and in children in particular. Similar to our results, a meta-analysis [7] demonstrated an increased rate of complicated appendicitis during the COVID-19 pandemic. This was explained by delayed presentation secondary to avoidance of hospital visits due to fear of exposure to the virus [12,13,14,15,16,17,18,19].

Humanitarian disasters in general may cause direct injuries and loss of life. Moreover, these hazards also manifest secondary health impacts [20]. Such situations are particularly critical when it comes to surgical care, because the human, material, and financial resources needed for surgical services are often the most difficult to recruit [21]. Civilians frequently suffer severe consequences as a result of armed conflicts [22,23]. Our study underscores the same effect on pediatric surgical patients under the unfortunate circumstances of a war conflict.

In this study, we demonstrated that the rate of complicated appendicitis doubled during the war in medical centers close to conflict zones. Interestingly, however, we found that time to presentation to the ED was similar before and during the war, even in medical centers close to conflict zones. This finding may indicate good organization of medical services, as well as moderate threats to the civilian population of Israel from the adverse consequences of the ongoing war. We found another study with similar findings (Ko et al., 2019) [24] that investigated possible associations between delayed transfer to a tertiary facility due to the military environment in South Korea and the outcome in patients who underwent an appendectomy. They did not find any correlation between the time of transfer (early vs. late) and the rate of appendicular perforation [24]. They postulated that their findings contribute to the evolving understanding that appendicitis does not require emergency surgery, and therefore, the absence of a difference between the groups could be attributed to the administration of antibiotics [24].

Another unexpected observation in our study was that the increase in complicated appendicitis was not reflected in longer LOS. This may be explained by the need of the medical centers to maintain vacancies for emergency admissions due to multiple casualty incidents that could occur, which may have had an influence on the early discharge of patients. Another aspect that may have influenced early discharge of patients may be parents’ preference to be at home with their other children, or feeling safer at home with or near a protected room or structure. In our study, we found no differences regarding postoperative complications, readmissions, or the rate of NOM and its failure between war and pre-war groups. In contrast to our findings, Delagdo-Miguel demonstrated increased LOS in children with AA treated during COVID-19 [18].

An assumption might arise that acute psychological stress inflicted by the intense war on populations living near conflict zones plays a role in the increased incidence of complicated appendicitis in these areas. The relationship between stress and its impact on inflammatory processes and infections is multifaceted and has been the subject of considerable research. Stress has been recognized as a significant risk factor for various diseases, including respiratory infections [24,25], asthma [26], and gastrointestinal disorders such as peptic ulcers and ulcerative colitis [26]. Stress has been shown to trigger neuroendocrine responses that can lead to immune dysregulation. Sustained elevated cortisol concentrations, which exacerbate inflammation, have been associated with chronic stress [27]. Stress-associated exacerbation or alteration of cytokine production has also been linked to the development of autoimmune diseases and impairment in the body’s ability to defend against infections [26,28]. Specific associations between psychosocial stress and gastrointestinal disease have been reported. Psychological stress has been linked to compromise of the integrity of the intestinal barrier, allowing commensal bacteria to penetrate the gastrointestinal mucosa, which can provoke inflammation and contribute to disease development [27], and low resilience to psychological stress has been linked to a higher likelihood of developing inflammatory bowel disease [27]. Anxiety and stress-related disorders, such as generalized anxiety disorder and panic disorder, have been associated with elevated inflammatory markers, including CRP [29]. Therefore, the trend of increased CRP levels in patients admitted to medical centers close to conflict zones during war, as demonstrated in our study, might link the significantly increased proportion of complicated appendicitis in this population to the postulation that stress is the reason for this increase. On the other hand, elevated CRP levels might merely serve as a marker for the severity of disease, since the severity of inflammatory diseases is typically characterized by elevated leukocyte count and CRP levels [30]. As highlighted by Kim et al., higher CRP levels correlate with more complicated forms of appendicitis [31,32].

Our findings highlight critical questions regarding the complex effects of intense war conflicts on healthcare delivery, patient behaviors, and disease severity. Additional research is needed to investigate the underlying drivers of these patterns and to identify effective strategies for improving patient care in conflict-affected areas.

This study has several limitations. First, as a retrospective study, it is more prone to incomplete data in medical records and to recall bias. Second, as a multicenter study, it is prone to variability of treatment and documentation throughout the different medical centers, which was not controlled. Furthermore, surgeon decision-making may differ among treating physicians. Although patient data were collected from several medical centers, we could not demonstrate statistically significant differences when analyzing the entire population. This is probably because the rates of complicated appendicitis in our population were lower than the rates reported in the literature for other populations. This leads to underpowered statistical comparisons. In addition, the division between “war” and “pre-war”, and the short period of time sampled, may not take in consideration the full complexity and fluctuations of the conflict (border areas were subject to intermittent rocket attacks before the war, whereas central areas of Israel were subject to rocket attacks during the war albeit less frequently than border communities) and the subsequent adaptations of the healthcare system. The short time examined evaluated only the most intense portion of the conflict, and the possible effects of chronic stress over a longer time period were not evaluated. In addition, a better adjustment of confounders such as socioeconomic status, healthcare access, or pre-existing regional differences might have clarified the data and results achieved. Future research with better solutions for these limitations, including evaluation over a longer time period, should be conducted in order to provide more validated conclusions.

## 5. Conclusions

An increase in complicated appendicitis was observed during the war in medical centers close to conflict zones, along with a trend toward increased CRP levels in this population. While the exact causes of these findings remain unclear, they highlight the complex impact of war on healthcare. As during war, medical centers are prepared and reinforce their trauma, orthopedic, and rehabilitation units, our data suggest that pediatric surgical services might need extra attention during war as well.

## Figures and Tables

**Table 1:** AAST appendicitis score [8].

AAST Grade	Description	Clinical Criteria	Imaging Criteria (CT Findings)	Operative Criteria	Pathologic Criteria
I	Acutely inflamed appendix, intact	Pain, leukocytosis, and right lower quadrant (RLQ) tenderness	Inflammatory changes localized to the appendix ± appendiceal dilation ± contrast nonfilling	Acutely inflamed appendix, intact	Presence of neutrophils at the base of crypts, submucosa ± in the muscular wall
II	Gangrenous appendix, intact	Pain, leukocytosis, and RLQ tenderness	Appendiceal wall necrosis with contrast nonenhancement ± air in the appendiceal wall	Gangrenous appendix, intact	Mucosa and muscular wall digestion; not identifiable on hematoxylin–eosin stain
III	Perforated appendix with local contamination	Pain, leukocytosis, and RLQ tenderness	As above with local periappendiceal fluid ± contrast extravasation	As above, with evidence of local contamination	Gross perforation or focal dissolution of the muscular wall
IV	Perforated appendix with periappendiceal phlegmon or abscess	Pain, leukocytosis, and RLQ tenderness; may have a palpable mass	Regional soft tissue inflammatory changes, phlegmon, or abscess	As above, with abscess or phlegmon in the region of the appendix	Gross perforation
V	Perforated appendix with generalized peritonitis	Generalized peritonitis	Diffuse abdominal or pelvic inflammatory changes ± free intraperitoneal fluid or air	As above, with the addition of generalized purulent contamination away from the appendix	Gross perforation

**Table 2:** Demographic and clinical characteristics of patients in the war group versus the pre-war group across all participating centers.

KERRYPNX	War; *n =* 106	Pre-War; *n =* 103	*p*-Value
**Gender**			0.093
Male	59 (56%)	69 (67%)	
Female	47 (44%)	34 (33%)	
**Age (years, mean ±SD)**	10.7 ± 3.8	11.4 ± 3.6	0.17
**Time to ED (hours, median [IQR]) **	24 (12–30)	24 (12–24)	0.64
**Time to ED ≥ 48 h**	25 (23.6%)	24 (23.3%)	0.96
**WBC (10 × 10^3^/µL)**	14.7 ± 4.9	15.0 ± 5.5	0.74
**ANC (10 × 10^3^/µL)**	11.1 ± 5.0	11.5 ± 5.1	0.63
**% Neutrophils**	76.1 ± 11.0	77.0 ± 12.7	0.62
CRP * (mg/L, median [IQR])	20.5 [4.0–58.1]	13 [2.5–36.1]	0.19
**AAST score**	1.43 ± 1.11	1.57 ± 1.13	0.39
**Complicated appendicitis**	28 (26.4%)	18 (17.5%)	0.11

AAST—The American Association for the Surgery of Trauma; ANC—absolute neutrophil count; CRP—C-reactive protein; ED—emergency department; *n*—number of patients; SD—standard deviation; WBC—white blood cell. * Non-normal distribution.

**Table 3:** Treatment and outcome of patients with AA in the war group versus the pre-war group across all participating centers.

	War; *n =* 106	Pre-War; *n =* 103	*p*-Value
**NOM for simple appendicitis**			0.43
Yes	20/104 (19.2%)	24/101 (23.8%)	
No	84/104 (80.8%)	77/101 (76.2%)	
**NOM failure**	4/19 (21.1%)	2/24 (8.3%)	0.38
**Length of stay (days, mean ±SD)**	3.30 ± 2.85	3.66 ± 2.58	0.35
**Readmission**	7/95 (7.4%)	8/88 (9.1%)	0.67
**Postoperative complications**	4/80 (5.0%)	2/75 (2.7%)	0.68

*n*—number of patients; NOM—nonoperative management; SD—standard deviation.

**Table 4:** Comparison of AA before and during the war in medical centers close to conflict zones.

	War; *n =* 42	Pre-War; *n =* 53	*p*-Value
**Gender**			0.61
Male	24 (57%)	33 (62%)	
Female	18 (43%)	20 (38%)	
**Age, years**	10.4 ± 4.2	11.5 ± 3.2	0.14
Time to ED, hours, median [IQR] *	24 (12–24)	24 (12–24)	0.32
**Time to ED ≥ 48 h**	5 (11.9%)	8 (15.1%)	0.65
**WBC (10 × 10^3^/µL)**	15.7 ± 4.8	15.5 ± 6.1	0.88
**% Neutrophils**	78.9 ± 8.8	78.4 ± 11.8	0.82
CRP * (mg/L, median [IQR])	26.5 (5.3–107.0)	13 (3.4–40.9)	0.075
**AAST score**	1.71 ± 1.13	1.40 ± 1.10	**0.18**
**Complicated appendicitis**	16 (38.0%)	9 (17.0%)	**0.016**
**NOM for simple appendicitis**			0.27
Yes	8 (19.0%)	15/52 (28.8%)	
No	34 (81%)	37/52 (71.2%)	
**Length of stay (days, mean ±SD)**	3.41 ± 2.6	3.51 ± 2.17	0.85

AAST—The American Association for the Surgery of Trauma; CRP—C-reactive protein; ED—emergency department; IQR—interquartile range; *n*—number of patients; NOM—nonoperative management; SD—standard deviation; WBC—white blood cell. * Non-normal distribution.

**Table 5:** Comparison of AA before and during the war in medical centers distant from conflict zones.

	War; *n =* 64	Pre-War; *n =* 50	*p*-Value
**Gender**			0.058
Male	35 (55%)	36 (72%)	
Female	29 (45%)	14 (28%	
**Age, years**	11.3 ± 3.9	10.8 ± 3.6	0.59
Time to ED, hours, median [IQR] *	24 (8.8–48)	20 (12–46)	0.91
**Time to ED ≥ 48 h**	8 (12.5%)	9 (18.0%)	0.41
**WBC (10 × 10^3^/µL)**	14.3 ± 5.0	14.6 ± 5.1	0.72
**% Neutrophils**	74.7 ± 11.8	75.9 ± 13.3	0.59
CRP * (mg/L, median [IQR])	19 (2–47.5)	13 (2.2–35.4)	0.59
**AAST score**	1.47 ± 1.12	1.46 ± 1.12	0.97
**Complicated**	12 (18.8%)	9 (18.0%)	0.92
**NOM for simple appendicitis**			0.89
Yes	12/62 (19.4%)	9/49 (18.4%)	
No	50/62 (80.6%)	40/49 (81.6%)	
**Length of stay (days, mean ±SD)**	3.23 ± 2.9	3.82 ± 2.9	0.30

AAST—The American Association for the Surgery of Trauma; CRP—C-reactive protein; ED—emergency department; IQR—interquartile range; *n*—number of patients; NOM—nonoperative management; SD—standard deviation; WBC—white blood cell. * Non-normal distribution.

## Data Availability

The raw data supporting the conclusions of this article will be made available by the authors upon request to interested researchers. Data are available on request only, due to privacy restrictions.

## Abbreviations

The following abbreviations are used in this manuscript:

AA	Acute appendicitis
AAST	American Association for the Surgery of Trauma
ANC	Absolute neutrophil count
CRP	C-reactive protein
CT	Computed tomography
ED	Emergency department
LOS	Length of stay
NOM	Nonoperative management
PAS	Pediatric appendicitis score
SD	Standard deviation
WBC	White blood cell
